# *Arabidopsis* Antiporter Genes as Targets of NO Signalling: Phylogenetic, Structural, and Expression Analysis

**DOI:** 10.3390/ijms26157195

**Published:** 2025-07-25

**Authors:** Rabia Amir, Zuhra Qayyum, Sajeel Hussain, Byung-Wook Yun, Adil Hussain, Bong-Gyu Mun

**Affiliations:** 1 Atta-ur-Rahman School of Applied Biosciences (ASAB), National University of Sciences & Technology (NUST), Islamabad 44000, Pakistan; 2 School of Applied Biosciences, College of Agriculture and Life Science, Kyungpook National University, Daegu 41404, Republic of Korea; 3 Department of Agriculture, Abdul Wali Khan University, Mardan 23200, Pakistan; 4 Department of Environmental and Biological Chemistry, Chungbuk National University, Cheongju 28644, Republic of Korea

**Keywords:** nitric oxide, antiporter, abiotic stress

## Abstract

Nitric oxide is a gaseous signalling molecule produced by plants. Slight changes in endogenous NO levels have significant biochemical and physiological consequences. We investigated the structural and functional properties of NO-responsive antiporter genes in *Arabidopsis thaliana*. Phylogenetic analysis of 50 antiporter genes classified them into four subgroups based on the presence of NHX and CPA domains and the evolutionary similarity of the protein sequences. Antiporters were found scattered across the five chromosomes with unique physico-chemical properties and subcellular localisation in the plasma membrane, nucleus, chloroplasts, and vacuole. Furthermore, we performed QPCR analysis of eight different antiporter genes after infiltrating the plants with 1 mM CySNO (S-nitroso-L-cysteine), a nitric oxide donor, in WT and the loss-of-function *atgsnor1-3* (disruptive S-nitrosoglutathione reductase 1 activity) plants. The *AT1G79400* (*CHX2*), *AT2G38170* (*RCI4*), and *AT5G17400* (*ER-ANT1*) showed a significant increase in their expression in response to CySNO infiltration. However, their expression in *atgsnor1-3* plants was found to be lower than in the WT plants, indicating a significant redundancy in the response of these genes to 1 mM levels of CySNO and physiological levels of SNOs in *atgsnor1-3*. On the other hand, a significant reduction in the expression of *AT1G16380* (*CHX1*), *AT2G47600* (*MHX1*), *AT3G13320* (*CAX2*), and *AT5G11800* (*KEA6*) was observed in WT plants after CySNO infiltration as well as in the leaves of *atgsnor1-3* plants. Our study identified three NO-responsive antiporter genes in *Arabidopsis*, indicating their roles in stress responsiveness and ion homeostasis that could be used for further validation of their roles in NO signalling in plants.

## 1. Introduction

Plants are constantly exposed to variable environmental circumstances and have evolved advanced processes to adjust their cellular metabolism proportionately.

These include strict regulation of the influx and efflux of various ions across the membranes by employing several membrane ion transporters such as cation/proton antiporters [1]. Cation proton antiporters (CPAs) are essential membrane proteins that regulate the transport of essential ions such as Na+ and H+, K+, and Li^+^ across the membrane [2], playing an essential role in plant growth and development, ion homeostasis, pH regulation, and abiotic stresses. Cation proton antiporters are classified into two superfamilies: CPA1 and CPA2 [3]. Na^+^/H^+^ antiporters, which belong to the NHX superfamily of cation transporters (CPA1), are involved in regulating the cell expansion salt tolerance, storing ions in the vacuole, and controlling the low Na^+^ concentration during salt stress [4].

Ca+/H+ exchangers that transport calcium and the cation diffusion facilitator (CDF) proteins involved in the transportation of metal ions such as cadmium, iron, and zinc are two other important families of antiporters. The cation diffusion facilitator family (CPA2) proteins are also thought to be involved in the transport of nickel, copper, and mercuric ions [5,6]. Furthermore, K+ influx transporters are members of the CPA superfamily [3].

Nitrate is an essential nitrogen nutrition source for plants and serves as fertiliser in agriculture. Most plants utilise two different types of nitrate transporters to transport nitrate (NO_3_)^1−^ actively, which do not share sequence similarity; however, they possess the same membrane domain, mostly localised in the plasma membrane. However, recently, *AtNRT2.7* has been identified to be found in the tonoplast membrane [7]. The nitrate transporter 1 family consists of 53 members, while NRT2 has seven members [8] with different localisation and functions, such as nitrate transport in vascular bundles, regulation of nitrates in seeds, and storage of nitrates in vacuoles [9]. Notably, the NRT2.1 is one of the main nitrate transporters in *Arbidopsis thaliana*, functioning at low nitrate concentration and is also involved in the initiation of lateral root nitrate uptake [10] while the expression of NRT2.4 is upregulated in the lateral roots of *Arabidopsis* plants under deprived nitrate concentration [11], emphasising the role of antiporters in nitrate signalling.

Nitric oxide is a diatomic gaseous signalling molecule endogenously produced in plants, controlling a wide range of biological processes. Slight variations in the level of NO or any of its various adducts, also known as reactive nitrogen intermediates (RNIs), have significant biochemical consequences at the cellular level. In animals, NO is produced enzymatically via three isoforms of the canonical nitric oxide synthase (NOS) enzyme. After decades of research, it is now clear that in plants, there is not one but several routes for NO production. Nitric oxide is a highly reactive free radical, allowing it to interact with reactive oxygen species (ROS), transition metals, allowing it to regulate homeostasis and signalling [12]. Perturbed levels of NO accumulation in plants are an important phenomenon affecting plant development and responses to various stresses [13]. Apart from regulating responses to abiotic and biotic stresses, NO is an important regulator in several processes, including germination of seed [14], photomorphogenesis [15], flowering [16], fruit ripening [17], and leaf senescence [18], acting as a key factor regulating the stress-responsive signalling pathways [19].

Several studies have shown the significant role of NO as a secondary messenger contributing to enhancing salt tolerance by enhancing the activation of ion transporters [20]. For instance, Nitric oxide promotes salt tolerance in wheat roots by significantly upregulating the expression of plasma membrane H^+^-ATPase and pyrophosphatase (PPase) [21]. It improves plant photosynthetic ability by protecting leaf pigments. The results were observed in *Solanum melangena* when photosynthesis was increased with yield enhancement of photosystem II (PSII) as a result of NO treatment. Another study reported that maize seedlings showed resistance by upregulating H+-ATPase and Na+/H+ antiporters in the tonoplast to increased salt content when exogenous NO was applied to the seedlings [22].

A combined treatment of NO and H_2_S on barley seedlings resulted in increased uptake of Na^+^/H^+^ by the enhanced expression of *HvAKT1* and *HvAKT4*, *HvNHX2*, *HvHVA-β*, *HvHA1*, and *HvSOS1*, improving tolerance to salinity [23]. Similarly, NO facilitates K^+^/Na^+^ balance in the roots of mangrove plants by increasing the expression of AKT1-Type K+ channel and Na+/H+ antiporters. Apart from stress regulation, members of the cation proton transporters are also involved in a range of developmental processes, such as the NHX in cell expansion [24], pH regulation [25], salt stress [26], K+ balance, transporting Na+ and cellular vesicle trafficking [27]. At*CHX14* effectively regulates the K+ concentration and is located on PM [28]. At*CHX21* increases salt tolerance in *Arabidopsis* [29] as well as coordinating with At*CHX23* in guiding pollen to ovules [30]. Pbr*CHX16* has been identified to play a significant role in the growth of pollen tubes [31]. GmKEA2 is actively involved in nodulation [32] while VvNHX1 is expressed during fruit ripening [33].

Increasing research suggests the role of antiporters in salinity and drought, where changes in the level of nitric oxide regulate the expression of antiporter genes. These studies suggest that antiporters may be directly or indirectly involved in nitric oxide signalling. This prompted us to characterise NO-response antiporter genes in *Arabidopsis thaliana* to investigate their potential role in downstream NO signalling. This research aims to characterise the structural and functional properties of NO-regulated antiporter family members in the model plant *Arabidopsis* and identify potential candidates involved in NO signalling.

## 2. Results

### 2.1. Identification of Genes and Phylogenetic Analysis

**Figure 1:**
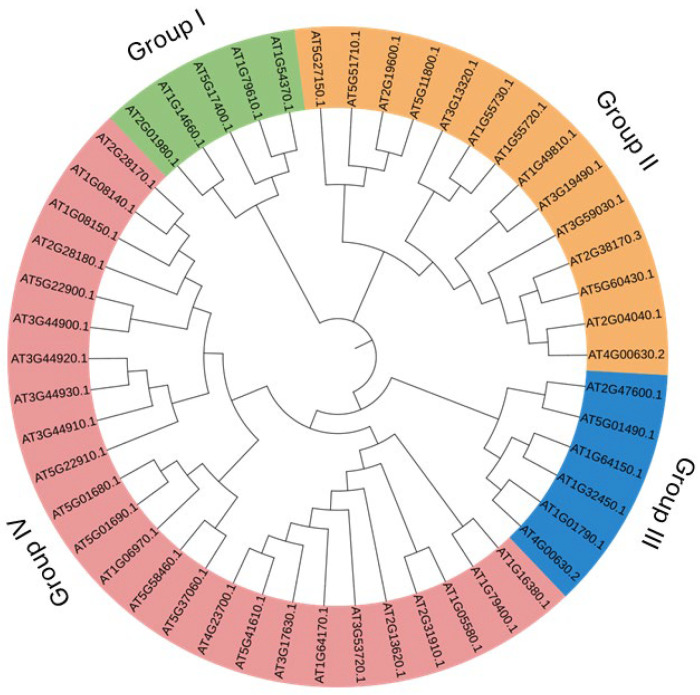
Phylogenetic analysis of the antiporter genes. Predicted amino acid sequences were aligned using MUSCLE, and a phylogenetic tree was created, keeping a 1000 bootstrap value. The different colour codes represent four antiporter groups (I, II, III, and IV) in which these genes were distributed.

A simple sequence-based and keyword (antiporter)-based search on the TAIR (https://www.Arabidopsis.org/, accessed on 26 March 2024) database yielded around 50 antiporter genes. Phylogenetic analysis of the 50 antiporter genes classified them into four subgroups (I, II, III and IV) based on the evolutionary similarity of the protein sequences (Figure 1). While the main classification of antiporter genes is based on the presence of NHX and CPA domains, evolutionary analysis gives an idea about the evolution of genes at different times in history. Several isoforms 104 were present in the antiporter genes, such as *CHX2* (*AT1G79400.1*) and *CHX1* (*AT1G16380.1*) showed a close evolutionary relationship. In addition, *CHX26* (AT5G01680.1) and *CHX27* (AT5G01690.1), CHX7 (AT2G28170.1) and CHX6A (AT1G08140.1), *CHX23* (AT5G22900.1) and CHX4 (AT3G44900.1), *CHX11* (AT3G44920.1) and *CHX10* (AT3G44930.1), *CHX25* (AT5G58460.1) and *CHX24* (AT5G37060.1), *CHX21* (AT2G31910.1) and *CHX23* (AT1G05580.1), KEA1 (AT1G01790.1) and KEA2 (AT4G00630.2), NHX6 (AT1G79610.1), and NHX5 (AT1G54370.1), KEA4 (AT2G19600.1) and *KEA6* (*AT5G11800.1*), CAX6 (AT1G55720.1), and CAX5 (AT1G55730.1) also showed close evolutionary relationship. On the other hand, CHX7, CHX6A, *CHX23*, *CHX24*, *CHX11*, *CHX10*, *CHX25*, *CHX24*, *CHX26*, and *CHX27* were clustered in one group (Figure 1). The phylogenetic analysis distributed 25 genes in the CHX subgroup, six genes each in KEA and CAX subgroups and five genes in the NHX group.

### 2.2. Domain Analysis

**Figure 2:**
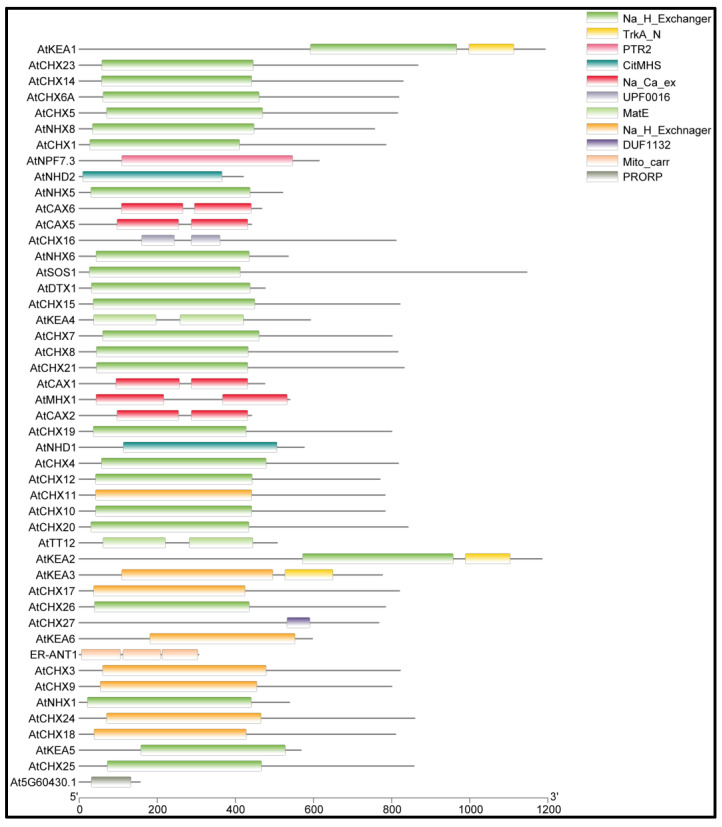
The detailed diagram of conserved domains in the antiporter proteins. The *x*-axis is showing the length of the protein, while the boxes represent the location of the conserved domain in the protein. AT*CHX27* protein contained domain DUF1132 with an unidentified function.

The sequence analysis of queried genes in Inter-ProScan identified various NHX and CPA domains among the 50 *Arabidopsis* antiporter genes (Figure 2). The identified domains include IPR030153 (CHX), IPR018409 (NHX), IPR018422 (CPA1), IPR000109 (Oligopeptide transporter), IPR004709 (NHX), IPR004713 (CAX), IPR004798 (CAX), IPR001727 (Gdt1 family), IPR018422 (CPA1), IPR004709 (NHX), IPR002528 (Multiantimicrobial extrusion protein), IPR002067 (Mitochondrial carrier protein), and IPR002113 (ADP/ATP carrier protein). Domain analysis showed that the Na_H_Exchanger domain is conserved across most of the proteins (Figure 2). One of the antiporter proteins, AT*CHX27*, contains a DUF1132 domain whose function is still unidentified.

### 2.3. Chromosomal Distribution of Genes

**Figure 3:**
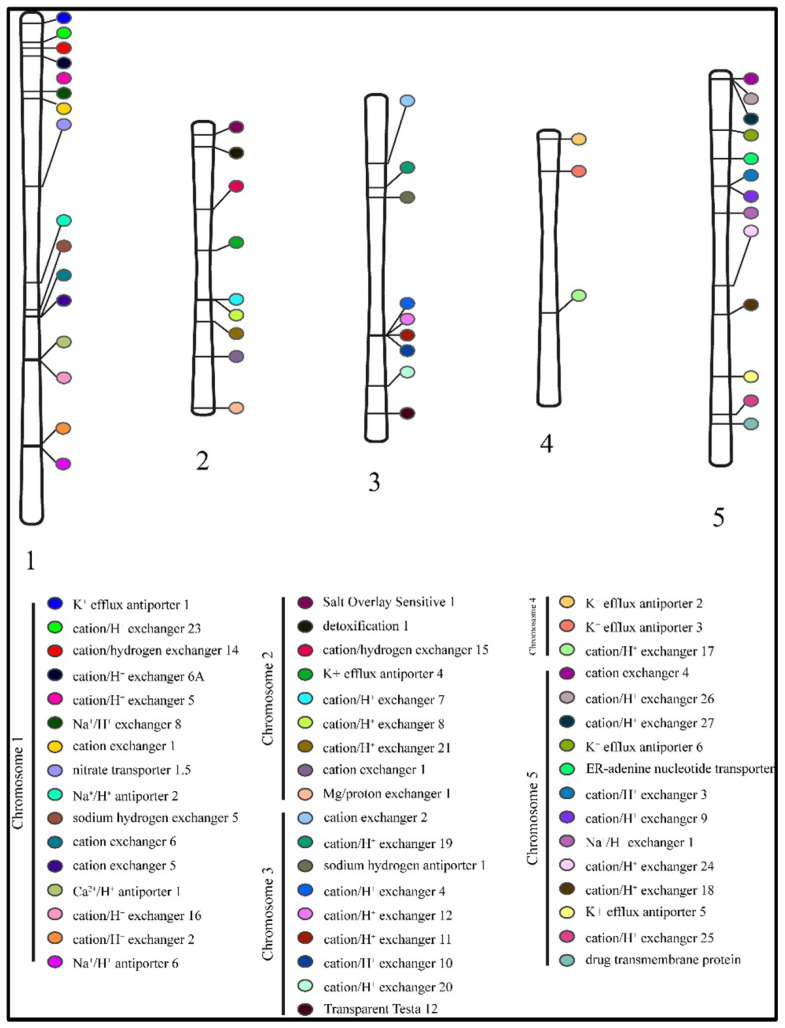
Distribution of 50 antiporter genes on the five chromosomes. The colour code denotes the respective member of the antiporter family.

The location of genes on chromosomes showed a highly scattered distribution of the antiporter genes on chromosomes 1 to 5. A total of 16 genes were located on chromosome 1, and nine genes each on chromosomes 2 and 3. On the other hand, chromosome 4 expressed only three antiporter genes, whereas 13 genes were located on chromosome 5 (Figure 3).

### 2.4. Physiochemical Attributes of Antiporter Genes

Detailed physicochemical properties of the antiporter genes have been summarised in Table 1. The length of the predicted proteins ranges from the smallest, comprising 153 amino acids, to the largest, comprising 1193 amino acids, with a corresponding molecular weight of 17kDa and 128kDa, respectively. The variation in length of protein suggests the variable role based on the presence of different functional domains, regulatory regions, signal peptides, and additional transmembrane. For instance, the members of the NHX family have a longer length due to the presence of C-terminal regulatory tails. The wide range of calculated isoelectric point (pI) for the proteins indicates their diverse role under varying pH conditions. The grand average of hydrophobicity values determines the hydrophobic or hydrophilic nature of the proteins, and their values range between −2 and +2. For the *Arabidopsis* antiporters, the values were consistently negative and ranged between −0.042 and −0.823, suggesting the proteins to be hydrophilic. However, some proteins showed higher GRAVY values because of their role as membrane-specific proteins. Predictive protein localisation studies showed that a maximum of 40 out of 50 proteins were localised in the plasma membrane, aligning with their function of ion transport across the membrane. Interestingly, seven of the 50 proteins were predicted to localise in multiple subcellular locations, such as the plasma membrane, vacuole, cytoplasm, chloroplasts, and endoplasmic reticulum. for example, AtNHD1 and PAM71 are localised on the plasma membrane as well as the chloroplast, while AtCAX1, At*CAX2*, and AtCAX5 are localised to the plasma membrane and vacuole. The presence of transmembrane helices in the antiporter genes identifies their role as membrane transporters. 10–12 transmembrane helices were observed in 82% of the antiporter genes. The HMM search did not identify any transmembrane helix in At*KEA6*. These findings have provided functional diversity of the *Arabidopsis* antiporter genes and their potential role in membrane targeting. The predominantly membrane localisation and hydrophobicity indicate their role in transmembrane ion exchange and membrane signalling. Further study on their role in ion transport is required to understand their function better.

### 2.5. Motif Composition and Gene Structure Analysis

**Figure 4:**
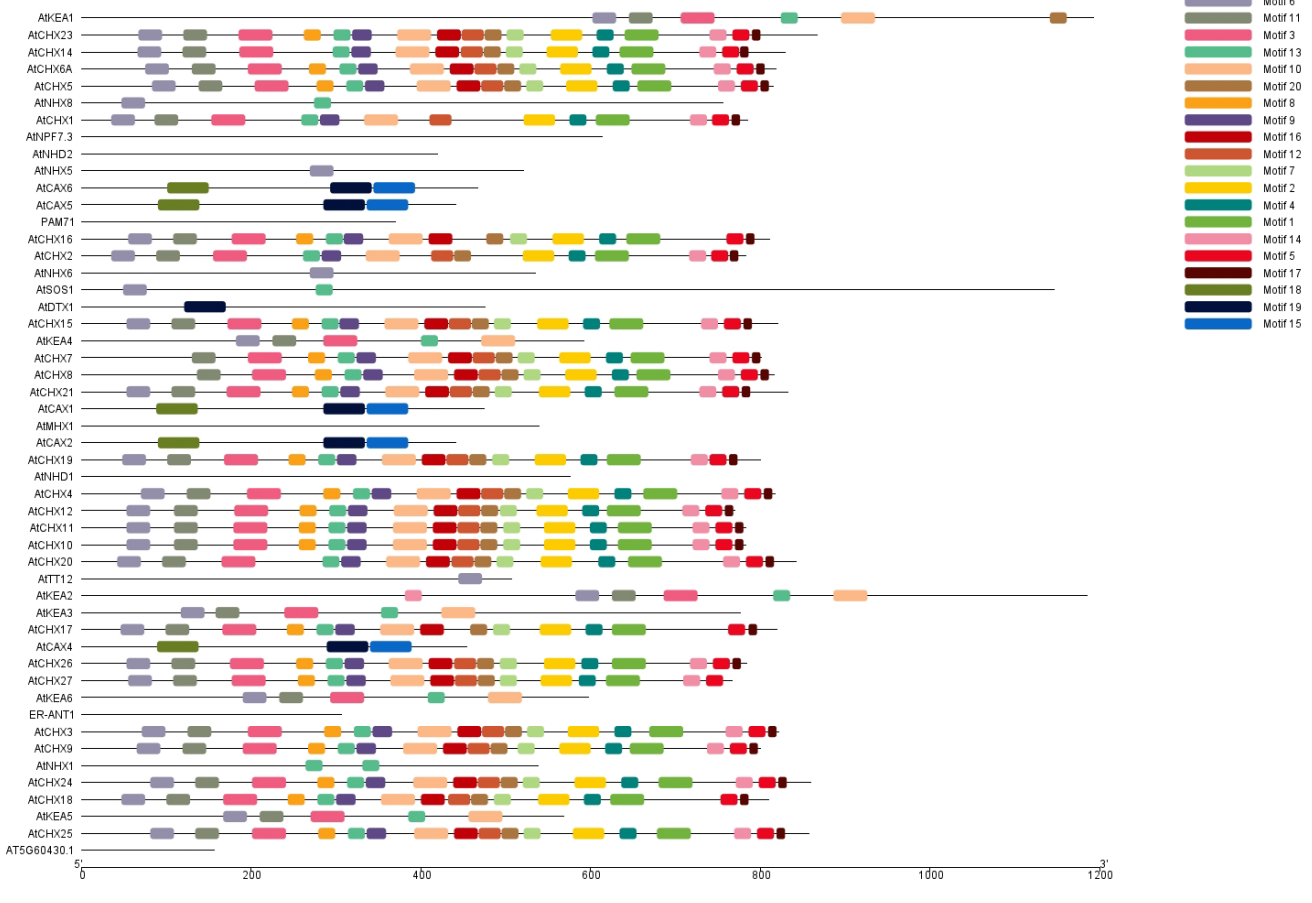
Motifs of the antiporter proteins were analysed using MEME, while the motif structure of proteins was drawn using TBTools. Twenty motifs were identified in the 50 antiporter proteins. Proteins which belonged to the same family reported the maximum number of conserved motifs in their sequence.

Motifs are small conserved amino acid sequence residues that play key structural and functional roles in a protein. The 50 antiporter *Arabidopsis* proteins were analysed for the presence of conserved motifs using MEME (https://meme-suite.org/meme/, accessed on 24 April 2024). We found 20 highly conserved motifs, which we labelled motif 1 through motif 20. These motifs were predominantly distributed among proteins belonging to the same subfamily, indicating conservation of the motifs. In the CHX and CPA families of antiporter genes, several motifs were identified to be preferentially distributed. The conserved motifs 1,2, 3, 4, 5, 6, 7, 8, 10, 11, 12, 13, 14, 16, 17, and 20 were found in the CHX family. The KEA subgroup of antiporter genes has conserved motifs 14, 6, 11, 3, 13, and 10. The motifs 1–8, 10, 13, 14, 16, and 17 were the most detected motifs that were conserved across the greatest number of proteins (Figure 4). In addition, motifs 18, 19, and 20 were shown to be conserved and dispersed across the CAX genes. However, none of the 20 conserved motifs were identified in *ER-ANT1*, AtNPF, AtNHD2, and AT5G60430, possibly indicating divergence in their function or evolutionary origin. The presence of conserved motifs across the different genes indicated similarity in their function as ion transporters and subcellular localisation.

**Figure 5:**
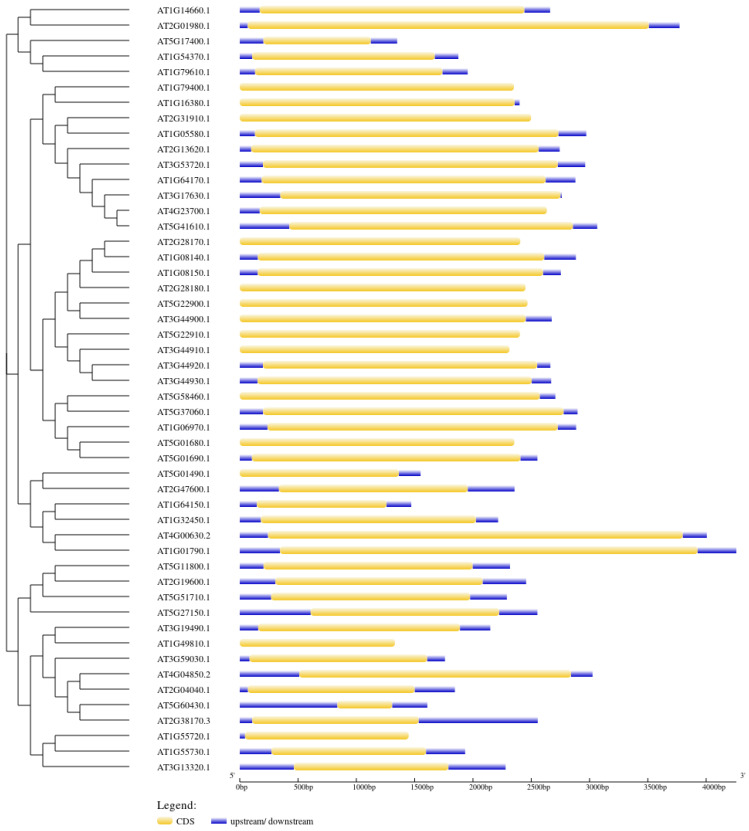
The exon–intron junction of the 50 *Arabidopsis* antiporter genes. The blue box represents the upstream and downstream regions of the gene. The yellow box represents the CDS, while the intron is represented by a black solid line.

The intron–exon junctions and structural composition of the 50 antiporter genes were analysed using Gene Structure Display (http://gsds.gao-lab.org/, accessed on 20 April 2024) (Figure 5). The analysis showed that the genes consist of a single intronless coding region. The genes had a complex structure with varying 5’ and 3’ UTRs and exon sizes (Figure 4). The length of exons for various subfamilies was different from each other, while they were quite similar within the same subfamily.

### 2.6. Cis-Elements Regulatory Analysis

**Figure 6:**
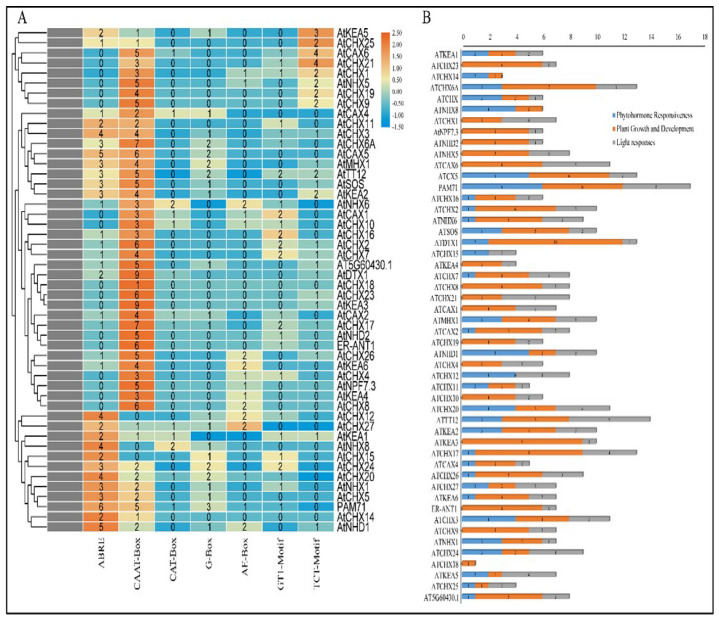
Cis-acting regulatory element analysis of the (**A**) promoter region of 50 antiporter genes showed that several genes contain regulatory elements involved in (**B**) light responsiveness, plant growth and development, and phytohormone responsiveness.

To identify putative *cis*-regulatory elements, a 1500 bp sequence upstream of the transcriptional start site of each antiporter gene was analysed in PlantCARE (https://bioinformatics.psb.ugent.be/webtools/plantcare/html/, accessed on 20 April 2024). The 50 *Arabidopsis* antiporter genes contained a total of 408 cis-regulatory elements (Figure 6A). The elements involved in abscisic acid responsiveness (ABRE), cis-elements in promoter and enhancer regions (CAAT-box), elements involved in meristem expression, light responsive (G-box, TCT-motif), and the AE-box motif involved in light responsiveness were among the cis-regulatory elements involved in stress response. Abscisic acid is a key regulator of a variety of signalling pathways involved in plant growth and development. Antiporter function and expression have been examined in a variety of plants, indicating that antiporters function under the impact of ABA. The presence of ABRE regulatory elements in 66% of the antiporter genes suggests ABA as a major hormonal regulator of antiporters. While CAAT-box promoter and enhancer elements are required for general transcription, CAT-box regulates meristem-specific expression of antiporters. On the other hand, the presence of light-responsive motifs predicts their potential regulation by light. The study predicted 15 CAT-box elements, 30 G-box elements, 23 AE-box motifs, 32 GT-1 motifs, and 38 TCT motifs in the 50 antiporter genes. The gene regulatory sequence analysis predicted their potential role in hormonal signalling and light responsiveness that can be confirmed through functional studies (Figure 6B).

### 2.7. Homology Modelling

**Figure 7:**
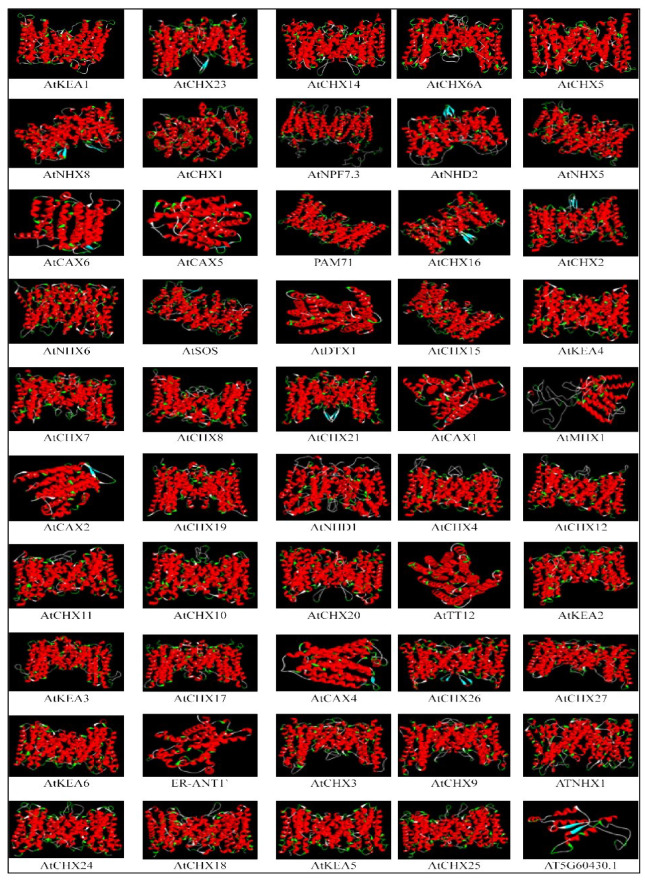
Predicted 3D models of *Arabidopsis* antiporter. The models were generated using SWISS-MODEL and visualised using Discovery Studio software version 24.1. Models were selected based on sequence coverage and sequence identity to the available template.

SWISS MODEL was used to create a 3D model of 50 antiporter gene family members, which is shown in Figure 7. The predicted models were chosen based on their similarity to the queried models in terms of sequence identity, coverage alignment, and confidence score. The entire model was made up of helices, with no evidence of a beta sheet in the structures. ATCAX5, ATCAX6, ATDX1, ATCAX1, AT*MHX1*, AT*CAX2*, ATTT12, ATCAX4, *ER-ANT1*, and the protein for AT5G60430 1 protein were monomeric, consisting of a single chain, while the remaining proteins were dimeric, consisting of two chains. Ramachan dran plot analysis was used to assess the quality of protein structure. The percentage of residues located in the most preferred region ranged from 81 percent to 97 percent for all antiporter structures, providing confidence in the predicted structure of antiporter proteins. The tertiary structure showed similarity among most antiporters, which suggests that these proteins may have evolved from the same ancestral gene sequence.

### 2.8. Gene Expression Analysis

**Figure 8:**
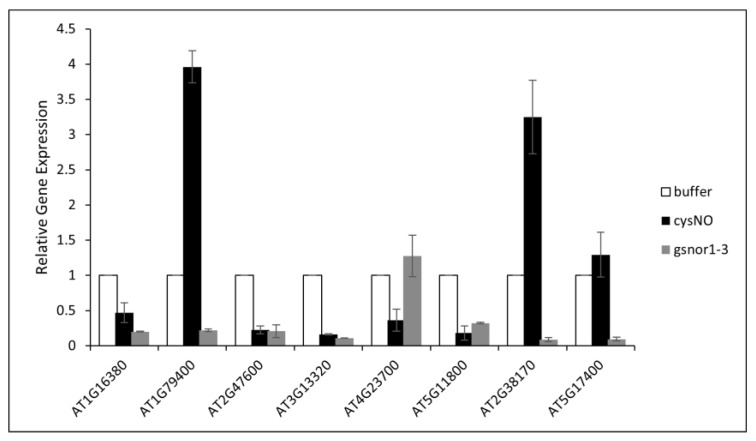
Expression analysis of antiporter genes to CysNO treatment in *Arabidopsis thaliana*, and *atgnsor1-3* plants. The plants were treated with a 1mM solution of CysNO while control plants were treated with buffer. Three biological replicates were used for each treatment, and Actin2 was used as a reference gene to normalise the expression analysis. The study identified three potential antiporter genes that might be involved in nitric oxide signalling.

Following the in-silico characterisation of nitric oxide-responsive antiporter genes in *Arabidopsis thaliana*, we performed quantitative real-time PCR analysis of eight different antiporter genes after infiltrating the plants with 1 mM CySNO as described by Hussain, Mun, Imran, Lee, Adamu, Shahid, Kim, and Yun [34]. CysNO is a small molecule used to stimulate NO signalling in plants. It is a NO donor and transfers NO to the thiol group through transnitrosylation during post-translational modification of protein, affecting protein function and stability. It is used in experiments in plants to study NO signalling during abiotic stress tolerance, defence activation and hormonal crosstalk [35]. Gene-specific primers were designed, the detail of which is given in Table 2. Furthermore, we also included the loss-of-function *atgsnor1-3* plants that naturally accumulate significantly higher levels of S-nitrosothiols (SNOs). The *atgsnor1-3* mutant has disrupted S-nitroso glutathione reductase activity, leading to the accumulation of nitrosoglutathione and altered NO signalling [34,35,36]. The *AT1G79400* (*CHX2*), which encodes a putative Na^+/^H^+^ antiporter family protein, *AT2G38170* (*RCI4*), encoding a high-affinity vacuolar calcium antiporter and *AT5G17400* (*ER-ANT1*), encoding an ER-localised adenine nucleotide transporter, showed a significant increase in their expression in response to CySNO infiltration. The expression was observed to be threefold higher than in control plants and 3.8-fold higher than the *atgsnor1-3* mutant plants. However, their expression in *atgsnor1-3* plants was found to be lower than in the WT plants, indicating a significant redundancy in the response of these genes to 1 mM levels of CySNO and physiological levels of SNOS in *atgsnor1-3* (Figure 7). On the other hand, a significant reduction in the expression of *AT1G16380* (*CHX1*), which encodes a putative Na^+^/H^+^ antiporter family protein, *AT2G47600* (*MHX1*), encoding a magnesium/proton exchanger, a member of the putative Na^+^/Ca^2+^ antiporter gene family, *AT3G13320* (*CAX2*) encoding a low affinity calcium antiporter. and *AT5G11800* (*KEA6*), which encodes a member of the putative potassium proton antiporter, was observed after CySNO infiltration as well as in the leaves of *atgsnor1-3* plants (Figure 8).

## 3. Discussion

Antiporter proteins are important regulators of ion homeostasis, plant growth, intracellular pH, defence genes, and production of reactive oxygen species during pathogen attack [37,38]. Given the large role of the antiporter gene family encoded in the plant genome, it is very likely that several members of the family contribute to NO-regulated physiological processes. However, the variable expression pattern and functional redundancy of these genes make it hard to predict their roles based simply on the similarity of sequences. To explore the role of antiporter genes in NO signalling, we performed a genome-wide analysis of the gene family in *Arabidopsis thaliana*, beginning with the identification of candidate genes, phylogenetic analysis, physicochemical characterisation, followed by expression of genes to CysNO treatment.

The study identified 50 antiporter genes in *Arabidopsis* and classified them into five major subfamilies based on the presence of conserved NHX, CHX, CAX, and KEA domains. The genes exhibited variable structural diversity, with most of the proteins predicted to have 10–12 transmembrane helices. Transmembrane domains are a part of the antiporter proteins that help them in their function of ion transport.

Several antiporter genes were predicted to have NHX and CHX domains. While a few have domains involved in transporting potassium ions across the membrane.

Motif analysis predicted the presence of conserved motifs across most of the antiporter genes, with a few exceptions where the proteins did not show any conserved motif. Several cis-regulatory elements were identified on the promoter region, predicting their role in plant growth and development, light responsiveness, and phytohormone signalling, emphasising their diverse role. Although antiporters are well studied for their role in stress responsiveness and ion transport, their role in NO signalling requires little consideration. The increased expression of three antiporter genes *AtCHX*, *RCl4*, and *ER-ANT1* to CysNO treatment predicts their role in NO signalling and ion homeostasis. The expression of *RCl4* needs further study based on its location in the vacuole, and a Ca^2+^ transporter suggests a role in NO-Ca^2+^ signalling similar to that observed in animals [39,40].

However, in plants, the mechanism of action of NO on Ca^2+^ signalling is still unclear, and studies are in process to completely understand the phenomenon. Several regulatory elements involved in plant growth and development, as well as light responsiveness, were found in At*CHX2* and *RCI4* (AtCAX1). The identification of these regulatory elements suggests that the NO signalling traverses with the wider stress and developmental signalling networks, a role that aligns with the multifactional nature of NO and antiporters. The expression of these genes, except At*CHX17* (Cation exchanger), was downregulated on CysNO treatment in *atgsnor1-3* plants. These results contrast with the reported function of SNOR genes, which actively regulate defence responses that need further comprehensive studies. The studies at the transcriptional level can provide further insight into the regulation and unusual response of *Atgsnor1-3* plants to CysNO treatment.

The study proposes potential antiporter genes involved in NO signalling, particularly those linked to Ca^2+^ and K^+^ exchange. Further research involving gene knock-out studies, ion leakage measurement, and localisation studies can provide a better insight into the role of these antiporters in NO signalling.

## 4. Materials and Methods

### 4.1. Identification of Antiporter Gene in Arabidopsis

The antiporter gene sequences in *Arabidopsis* were retrieved from NCBI and TAIR. The keyword “Antiporter” was used to search genes in the database. The retrieved sequences were then submitted to InterProScan (https://www.ebi.ac.uk/interpro/result/Inter-ProScan, accessed on 16 February 2024) to identify the NHX and CPA domains [41]. In order to confirm the NHX and CPA domains, the sequences were submitted to the SMART database (https://smart.embl.de/, accessed on 16 February 2024) [42]. Moreover, the transmembrane helices of the antiporter genes were identified by submitting the sequences to the TMHMM 2.0 server (https://services.healthtech.dtu.dk/service.php?TMHMM-2.0, accessed on 16 February 2024) [43].

### 4.2. Phylogenetic Analysis and Characterisation of Antiporter Proteins

In order to identify similarity and evolutionary relationship among the genes, phylogenetic analysis was performed, which also classified the antiporter genes into subfamilies within *Arabidopsis*. MEGA10 software was used to construct an unrooted phylogenetic tree using the Neighbour Joining method, creating the bootstrap replicates up to 1000. The tree was further modified using iTOL [44]. A gene structure display server was used to identify the coding region of antiporter genes.

### 4.3. Conserved Motif Analysis, Physiochemical Characterisation of Antiporter Genes

Multiple Em for Motif Elicitation (MEME) (version 5.5.4) (https://meme-suite.org/meme/tools/meme, accessed on 16 February 2024) [45] was used to identify conserved motifs in the antiporter genes. The parameters were set at the default, unless the number of motifs was adjusted to 20. The pattern of motif was drawn using TBtool software (https://github.com/CJ-Chen/TBtools-II, accessed on 16 February 2024). The physiochemical and molecular characterisation of proteins was performed using ExPASY Protparam (https://web.expasy.org/protparam/, accessed on 16 February 2024). This includes the isoelectric point pI, molecular weight, instability index, and hydrophilic or hydrophobic character of proteins. Moreover, the subcellular localisation of protein was predicted using the WoLF PSORT (Horton et al., 2007 [46]) server for all 50 antiporters.

### 4.4. Chromosomal Distribution of Antiporter Genes

The start and end location of genes on chromosomes was obtained from the TAIR database and used to show the location of genes on the chromosomes. Ritchielab (https://visualization.ritchielab.org/phenograms/plot, accessed on 16 February 2024) was used to map the location of genes on chromosomes.

### 4.5. Cis-Element Regulatory Analysis and Homology Modelling

The Phytozome database was used to extract 1500 bp upstream regions of the transcription start site for the 50 genes. The promoter region was further analysed in the PlantCARE (http://bioinformatics.psb.ugent.be/webtools/plantcare/html/, accessed on 16 February 2024) database to identify cis-acting elements in the promoter region. The elements involved in stress responses were selected and analysed. In addition, the 3D model of the 50 antiporter genes was predicted using SWISS-MODEL based on sequence homology to the available templates.

### 4.6. Plant Growth and CysNO Treatment

*Arabidopsis thaliana* Col-0 and *atgsnor1-3* mutant plants were grown in controlled environmental rooms under 16 h light and 8 h dark at 18 °C. The leaves of 4-week-old plants were infiltrated with 1 mM CysNO (mixing equimolar HCl dissolved L-cysteine and a sodium nitrite solution) on the leaves’ abaxial surfaces. The control plants were infiltrated with buffer (1 mM HCl) only. The leaves were collected 6 h after infiltration and snap frozen in liquid nitrogen for RNA extraction and qPCR analysis.

#### Real-Time PCR Analysis

Real-time PCR was performed for eight different antiporter genes. For this purpose, leaf samples were collected from control and 1 mM CysNO-infiltrated plants as previously described by Hussain, Mun, Imran, Lee, Adamu, Shahid, Kim, and Yun [34]. Total RNA was extracted using Trizol reagent (Ambion Life Technologies, Austin, TX, USA). cDNA was synthesised from 2 µg total RNA using a DiaStarTM RT Kit (SolGent, Daejeon, Republic of Korea). A two-step real-time PCR reaction was performed using an EcoTM real-time PCR system (Illumina, San Diego, CA, USA) using 2× Quantispeed SYBR Kit (PhileKorea, Seoul, Republic of Korea) with 100 ng template DNA and 10 nM of each primer in a final volume of 20 µL. Real-time PCR was performed by polymerase activation at 95 °C for 2 min, followed by denaturation at 95 °C for 5 s and concurrent annealing and extension at 65 °C for 30 s. The *Arabidopsis* actin (*ACT 2*) gene and a no-template reaction were used as controls.

## 5. Conclusions

In this study, we identified 50 antiporter genes in *Arabidopsis thaliana* based on the presence of the NHX and CHX domains. The genes were further classified into subfamilies based on the presence of several other cation exchanger domains. The *cis*-regulatory elements predicted their role in hormonal signalling, plant growth and development and light responsiveness. The expression of the cation exchanger, cold inducible gene, and endoplasmic reticulum adenine nucleotide transporter genes was elevated. The study helped us to lay a foundation for the functional characterisation of these antiporter genes through overexpression or silencing to better understand their role. Moreover, this would also help us to regulate the plant responses towards sustainable development.

## Figures and Tables

**Table 1:** The gene accession numbers, amino acid length, molecular weight, and subcellular localisation of proteins of the antiporter proteins in *Arabidopsis thaliana*.

Sr. No.	Gene ID	Length	MW	pI	GRAVY	Aliphatic Index	TM	Subcellular Localisation
1	AT1G01790.1	1193	128,033.50	5.22	0.080	105.80	10	plas
2	AT1G05580.1	867	95,867.43	6.02	0.246	99.64	12	plas
3	AT1G06970.1	829	92,159.60	6.56	0.335	109.5	12	plas
4	AT1G08140.1	818	93,395.50	7.27	0.146	97.21	10	plas
5	AT1G08150.1	815	91,606.19	6.56	0.328	106.50	10	plas
6	AT1G14660.1	756	83,475.75	6.58	0.273	108.94	12	plas
7	AT1G16380.1	785	88,908.78	6.31	0.295	107.62	12	plas
8	AT1G32450.1	614	68,699.10	5.50	0.076	88.93	11	plas
9	AT1G49810.1	420	45,446.85	6.19	0.823	124.19	9	vacu
10	AT1G54370.1	521	57,335.14	4.98	0.444	96.89	10	plas
11	AT1G55720.1	467	51,837.90	5.44	0.530	113.58	10	plas
12	AT1G55730.1	441	48,096.18	4.86	0.563	114.54	11	Plas/vacu
13	AT1G64150.1	370	39,073.58	4.88	0.462	104.19	7	Plas/chlo
14	AT1G64170.1	811	88,050.60	8.82	0.404	111.36	10	plas
15	AT1G79400.1	783	88,204.04	6.39	0.290	108.15	12	plas
16	AT1G79610.1	535	59,314.39	5.68	0.377	97.48	9	plas
17	AT2G01980.1	1146	127,188.31	7.62	0.098	105.30	9	Plas
18	AT2G04040.1	476	51,844.92	7.88	0.703	119.62	12	Vacu/plas
19	AT2G13620.1	821	89,859.54	5.71	0.363	109.93	12	plas
20	AT2G19600.1	592	64,249.42	5.91	0.589	118.72	12	plas
21	AT2G28170.1	801	90,989.71	7.29	0.261	104.84	10	plas
22	AT2G28180.1	816	90,956.09	7.22	0.384	116.81	12	plas
23	AT2G31910.1	832	91,982.03	5.38	0.256	102.43	12	plas
24	AT2G38170.3	475	51,636.65	5.92	0.604	124.42	8	Plas/vacu
25	AT2G47600.1	539	59,792.38	5.84	0.413	111.41	10	plas
26	AT3G13320.1	441	48,215.38	4.72	0.573	114.10	10	plas/vacu
27	AT3G17630.1	800	86,915.25	8.76	0.370	112	12	plas
28	AT3G19490.1	576	61,368.48	5.49	0.528	112.26	8	Plas/chlo
29	AT3G44900.1	817	92,007.93	8.51	0.337	107.85	10	nucl
30	AT3G44910.1	770	85,819.50	6.02	0.381	115.16	13	plas
31	AT3G44920.1	783	88,432.71	6.16	0.406	118.35	11	plas
32	AT3G44930.1	783	88,143.29	6.09	0.397	118.10	11	plas
33	AT3G53720.1	842	91,553.09	8.93	0.276	110.20	10	plas
34	AT3G59030.1	507	55,147.11	8.36	0.745	120.18	12	plas
35	AT4G00630.2	1185	127,605.10	5.11	0.086	105.22	10	plas
36	AT4G04850.2	776	83,790.69	5.53	0.369	111.69	-	plas
37	AT4G23700.1	820	89,165.51	8.06	0.372	112.80	12	plas
38	AT5G01490.1	454	49,609.17	6.08	0.519	117.91	10	plas
39	AT5G01680.1	784	87,038.08	7.02	0.256	106.98	10	plas
40	AT5G01690.1	767	86,990.99	8.74	0.396	113.82	11	plas
41	AT5G11800.1	597	64,391.65	7.10	0.599	125.53	12	plas
42	AT5G17400.1	306	33,788.18	9.82	0.041	88.99	4	cyto/cyto_E.R
43	AT5G22900.1	822	92,452.77	7.50	0.277	101.50	11	plas
44	AT5G22910.1	800	89,059.65	7.90	0.359	118.41	11	plas
45	AT5G27150.1	538	59,513.42	6.73	0.458	106.71	12	vacu
46	AT5G37060.1	859	96,680.97	6.34	0.115	95.17	10	plas
47	AT5G41610.1	810	87,383.34	8.71	0.398	113.38	12	plas
48	AT5G51710.1	568	61,598.40	5.84	0.613	123.86	11	plas
49	AT5G58460.1	857	95,833.29	8.32	0.118	94.39	11	plas
50	AT5G60430.1	156	17,439.83	5.76	−0.114	78.65	-	chlo

**Table 2:** The gene-specific primers used for RT-PCR analysis.

Sr. No.	Gene	Primer Name	Sequence	Amplicon Size (bp)
1	*AT1G16380*	At*CHX1*_L1	ATTGTCGGAGACGCGGTGAC	177
		At*CHX1*_R1	CAGCGTCTGCATTCCGTTGC	
2	*AT1G79400*	At*CHX2*_L1	CGCGGTGCGATCTCTCTTGT	145
		At*CHX2*_R1	TGCCTCCATCTTCGTCGTGC	
3	*AT2G38170*	AT*CAX1*_L1	ACGGCGAAAGGATCGAGCAG	233
		AT*CAX1*_R1	CAAGGCTGACTGACGCCACA	
4	*AT2G47600*	AT*MHX1*_L1	GTCAGCCATCACTGCACGGT	218
		AT*MHX1*_R1	ACCGCTCCCCCATATTCCGT	
5	*AT3G13320*	At*CAX2*_L1	CAGCTGAACATGCAGGGGCT	115
		At*CAX2*_R1	ACGCAGAATGGGACCGCAAA	
6	*AT4G23700*	AT*CHX17*_L1	ATTTGCCAAAGCGCGGAACG	267
		AT*CHX17*_R1	CGCAAACGCTAACGCCTCAC	
7	*AT5G11800*	AT*KEA6*_L1	TCGCCTTTGCTTGTGCTGGA	280
		AT*KEA6*_R1	TCCGAACGTTTACCACCGCA	
8	*AT5G17400*	*ER-ANT1*_L1	CCTCTGCAGGCGTCATTGCT	192
		*ER-ANT1*_R1	CAAGCACTCCTGCTCCTGCT	

## Data Availability

The original contributions presented in this study are included in the article. Further inquiries can be directed to the corresponding author(s).
